# Strophanthus-Lanoline

**Published:** 1887-05

**Authors:** 


### STROPHANTHUS-LANOLINE.

The Editor of the *British and Colonial Druggist*, (W. Lascelles Scott, F.C.S.), says in the issue of February 12th, 1887: I have just made some experiments which illustrate, at one and the same time, the penetrating power of lanoline when applied to the skin, and the action of strophanthin upon the heart. With a mixture of two parts lanoline and one of petrogell, 1 per cent. of strophanthin was intimately mixed. Of this ointment, twenty grains was rubbed upon my left arm, the skin having been first freed from the normal oleaginous exudations by the use of a little weak ammonia and a soft cloth. The strophanthin ointment was allowed to remain on until my heart-beats (which very conveniently, have been abnormally high of late) were diminished in number from 103 to 100 per minute, which was the case eighteen minutes and a half from the commencement of the experiment. The pulse continued to recede until a minimum was reached at 94, some fifty-two minutes from the time the ointment was first applied, no further change occurring for several hours afterward.—*British Medical Journal.*

